# Transcranial direct current stimulation in the management of epilepsy: a meta-analysis and systematic review

**DOI:** 10.3389/fneur.2024.1462364

**Published:** 2024-11-11

**Authors:** Yujie Chen, Zhujing Ou, Nanya Hao, Hesheng Zhang, Enhui Zhang, Dong Zhou, Xintong Wu

**Affiliations:** Department of Neurology, West China Hospital of Sichuan University, Chengdu, Sichuan, China

**Keywords:** brain stimulation, transcranial electric stimulation, seizure, temporal lobe epilepsy, nerve stimulation, brain polarization, randomized controlled trial

## Abstract

**Background:**

Transcranial direct current stimulation (tDCS) has recently become a novel and non-invasive treatment option for refractory epilepsy. Previous systematic reviews have suggested that tDCS may be effective in treating epilepsy, this study presents the first meta-analysis on its effectiveness.

**Methods:**

We searched PubMed, Embase, Cochrane Library, and Web of Science for relevant randomized controlled trials (RCTs) from database inception to May 2024. The Cochrane risk of bias tool RoB2.0 was used to assess the risk of bias. Primary outcomes included changes in seizure frequency from baseline and the proportion of patients with a ≥50% reduction in seizure frequency.

**Results:**

Of the 608 studies initially identified, 14 were finally included. The pooled results from the random-effects model indicated that tDCS significantly reduced seizure frequency (WMD 0.41, 95% CI 0.24, 0.59). Further subgroup analysis revealed that tDCS significantly reduced seizure frequency in temporal lobe epilepsy, and seizure frequency was more alleviated in studies that had treatment sessions of fewer than 5 times, and followed up within 2 months' post-treatment. Only four studies provided data on patients with a ≥50% reduction in seizure frequency, showing no significant difference (RR 2.96, 95% CI 0.85, 10.32). In the systematic review, three studies analyzed cognitive function changes after tDCS treatment, but none reported significant improvements. The most common side effect during tDCS treatment was transient tingling, and no patients required additional life-support measures due to side effects.

**Conclusion:**

The current meta-analysis on available trials indicates that tDCS can effectively reduce seizure frequency in the short term and is well-tolerated. However, its impact on cognitive improvement in epilepsy patients requires further investigation.

**Systematic review registration:**

https://inplasy.com/inplasy-2024-6-0033/, identifier INPLASY202460033.

## 1 Introduction

Affecting 70 million people globally, epilepsy stands as the second most prevalent neurological disorder. The World Health Organization has recognized it as one of the five major neurological and psychiatric diseases targeted for prevention and treatment (1). In China, it is estimated that there are around 10 million people with epilepsy, with an annual increase of 400,000 new cases (2). The frequency of seizures and whether antiepileptic drugs are used in combination profoundly impact patients' quality of life and living costs (3).

Among epilepsy patients followed up in clinical settings, about one-third suffer from refractory epilepsy (4). Only a minority of those with focal epileptic lesions are potential candidates for surgical resection (5). The complication rate for epilepsy surgery is ~10%, with serious complications occurring in about 1.5% of cases (6), with postoperative neurological deficits a major factor contributing to patient dissatisfaction (7).

For patients with refractory epilepsy unsuitable for resective surgery, neuromodulation therapies can be implemented. Neurostimulation reduces epileptic activity by delivering electrical or magnetic stimulation to various anatomical targets. Traditional neurostimulation methods include open-loop vagus nerve stimulation (VNS), open-loop deep brain stimulation (DBS), and closed-loop responsive neurostimulation (RNS) (8). However, these invasive treatments come with risks such as intracranial hemorrhage and infection (9), and their execution requires a high level of expertise, limiting them to specialized clinical centers (10). Moreover, these treatments are costly, requiring long-term maintenance of equipment, and their cost-effectiveness is still under evaluation (11, 12).

Two predominant forms of non-invasive brain stimulation techniques are transcranial direct current stimulation (tDCS) and transcranial magnetic stimulation (TMS). Both use electromagnetic principles to non-invasively modulate cortical excitability in a targeted spatial and temporal manner. Using tDCS may modulate cortical excitability by inducing depolarization (anodal stimulation) or hyperpolarization (cathodal stimulation) of resting membrane potentials via low-intensity electrical currents conducted through scalp electrodes, thereby altering spontaneous neuronal excitability and activity (13, 71). Due to advantages such as minimal side effects, low cost, and ease of operation, and with some studies showing potential for sustained home-based therapy (14, 15). This technique has been under investigation as a promising add-on treatment for epilepsy. Fregni et al. conducted the first controlled clinical trial of cathodal tDCS for epilepsy in 2006, demonstrating that tDCS stimulation is safe and significantly reduces epileptiform discharges (16). Beyond epilepsy, tDCS has shown efficacy in chronic pain (17), cognition (18, 19), and psychiatric disorder (20, 21), suggesting potential therapeutic value for epilepsy comorbidities. Mechanistic exploration of tDCS effects continues in animal studies; in pilocarpine-induced epilepsy models, tDCS mitigates hippocampal CA1 neuronal damage, thereby improving cognitive function and potentially reducing seizure frequency and severity (22). Moreover, in pentylenetetrazol (PTZ)-induced epilepsy models, tDCS has been found to suppress post-seizure inflammation and enhance brain function (23). Additionally, it may promote long-term depression (LTD) and potentiation (LTP) for synaptic plasticity and induce dopamine release in the dorsal striatum, further enhancing learning and memory performance (24, 25).

To our knowledge, the most recent comprehensive systematic review on this topic was published in 2021 (26), including studies using tDCS stimulation for treating patients with epilepsy. In follow-up, 84% (21/25) of the included studies reported a reduction in seizure frequency, with no reports of serious adverse events, suggesting it is generally a safe and effective intervention. However, the studies included in this review exhibited low quality and substantial heterogeneity. Recently completed new randomized controlled trials (RCTs) prompt us to conduct a first-time meta-analysis based on RCTs and systematically review the literature on the effects of tDCS on neurocognitive functions, aiming to provide new insights for clinical decision-making. At the time of writing our manuscript, a new systematic review and meta-analysis (27) has been published, which found that active transcranial direct current stimulation treatment significantly reduced seizure frequency by 3.15 seizures per month compared to the sham group. However, we focused on the analysis of seizure reduction rate, instead of seizure reduction numbers, to minimize the differences of baseline seizure frequency. And we further reviewed the improvement in cognitive ability and psychological state in patients. In addition, temporal lobe epilepsy, the most common drug-resistant epilepsy, were chosen for subgroup to review the effects of electrical stimulation.

## 2 Materials and methods

This study was carried out based on the Cochrane Handbook for the Systematic Review of Interventions (for details, see at: http://training.cochrane.org/handbook) and the Preferred Reporting Items for Systematic Reviews and Meta-Analyses Statement (PRISMA) (28). The research protocol was published on INPLASY (INPLASY202460033). This study does not require ethical approval or patient consent.

### 2.1 Search strategy

Two researchers independently searched the PubMed, Embase, Web of Science, and Cochrane Library databases using a combined approach of Medical Subject Heading (MeSH) terms and free-text terms. Search terms included epilepsy (MeSH) or Epilep^*^ or seizur^*^; Transcranial Direct Current Stimulation (MeSH) or tDCS or t-DCS or brain polarization or galvanic stimulation; and randomized controlled trial (Publication Type [pt]) or controlled clinical trial (pt) or random^*^ or placebo (Supplementary material). Searches were conducted from database inception to May 10, 2024.

### 2.2 Inclusion and exclusion criteria

Inclusion criteria include:

- Study type: Randomized controlled trials or randomized crossover trials.
- Study language: English literature.
- Study subjects: Patients clinically diagnosed with epilepsy according to the International League Against Epilepsy (ILAE).
- Intervention method: The control group received sham stimulation, while the experimental group received cathodal tDCS treatment.
- Study outcomes must include at least seizure frequency.

Exclusion criteria include:

- Duplicate literature.
- Reviews, systematic reviews/meta-analyses, case reports, conference papers, animal experiments, letters, patents, and unrelated literature.
- Literature for which the full text cannot be obtained.
- Studies without seizure frequency as an outcome.

### 2.3 Literature screening and data extraction

Two researchers rigorously conducted literature searches according to predefined inclusion and exclusion criteria. All identified studies were managed using EndNote X9 software, where duplicates were removed. Titles and abstracts were screened for initial relevance, and full texts were retrieved for further assessment against the criteria for this systematic review. Basic information from selected studies was extracted and cross-checked, including title, first author, publication year, and country of origin. Data extracted also encompassed participant details (sample size, patient age, and gender), parameters (anode and cathode positioning, electric current dose in mA, electrode size in cm^2^, electrode type), treatment duration and follow-up time (sessions, time of follow-up), and outcome measures. Discrepancies in data extraction were resolved through consensus with a third researcher. When multiple publications from the same trial or cohort were identified, the original reports with all pertinent data elements were included. The intended outcomes include the means, standard deviation, and differences in interictal epileptiform discharges (IEDs) in electroencephalogram (EEG), seizure frequency, neuropsychological test scores, and brain network before and after stimulation. Additionally, it includes the number of participants achieving a seizure reduction of ≥50% and any adverse events. Attempts were made to obtain individual participant data (IPD) from all included studies by contacting corresponding authors. Studies without accessible IPD were analyzed using reported study-level data.

### 2.4 Quality assessment of studies

Two researchers (CYJ and OZJ) independently conducted methodological assessments of the included literature using the Cochrane Risk of Bias Tool 2.0 (29). Discrepancies were resolved by a third researcher, and consensus was reached through discussion. The assessment covered five domains: bias in the randomization process, bias due to deviations from intended interventions, bias due to missing outcome data, bias in outcome measurement, and bias in selective reporting of results. Each domain was evaluated for “low risk of bias,” “some concerns,” or “high risk of bias.” This standardized approach, facilitated by the official tool, enhances procedural and methodological rigor while minimizing subjective influences.

### 2.5 Outcome measures

The primary outcome measures included: Seizure Reduction: defined as the change in seizure frequency relative to baseline following tDCS treatment. Favorable Response: defined as the proportion of participants achieving a 50% or greater reduction in seizure frequency. Secondary outcome measures included. EEG results including IED number or frequency. Functional magnetic resonance Imaging (fMRI): Including functional connectivity and task-based imaging. Psychological tests includes assessment for one or more domains of cognitive functions and psychological state. Adverse Effects: Number of participants experiencing adverse events and specific types described in the literature.

### 2.6 Statistical analysis

Data extraction from the literature was independently conducted by two researchers and entered into StataSE16 (Stata Corp, College Station, TX, USA) for statistical analysis. For binary outcome variables such as response rates, data were entered as event numbers, and relative risk (RR) was used as the effect size. Continuous data, such as improvement rates, were entered in mean and standard deviation format, and the weighted mean difference (WMD) was used as the effect size, with both reported with 95% confidence intervals (95%CI). Heterogeneity among included studies was assessed using the inconsistency index (*I*^2^). *I*^2^ values < 50% indicate no significant heterogeneity among studies, warranting the use of a fixed-effects model for analysis. *I*^2^ values >50% indicate significant heterogeneity among studies, requiring the use of a random-effects model for analysis. To explore potential sources of heterogeneity, subgroup analyses were performed based on follow-up time (within 1 month or within 2 months), treatment duration (sessions ≤ 5 or >5), and epilepsy type (including only temporal lobe epilepsy patients). Sensitivity analyses were subsequently conducted. Publication bias among included studies was assessed using Egger's test. All results were considered statistically significant at *P* < 0.05. Secondary outcome measures were subjected to descriptive analysis and reported in the systematic review.

## 3 Results

### 3.1 Literature retrieval process

**Figure 1:**
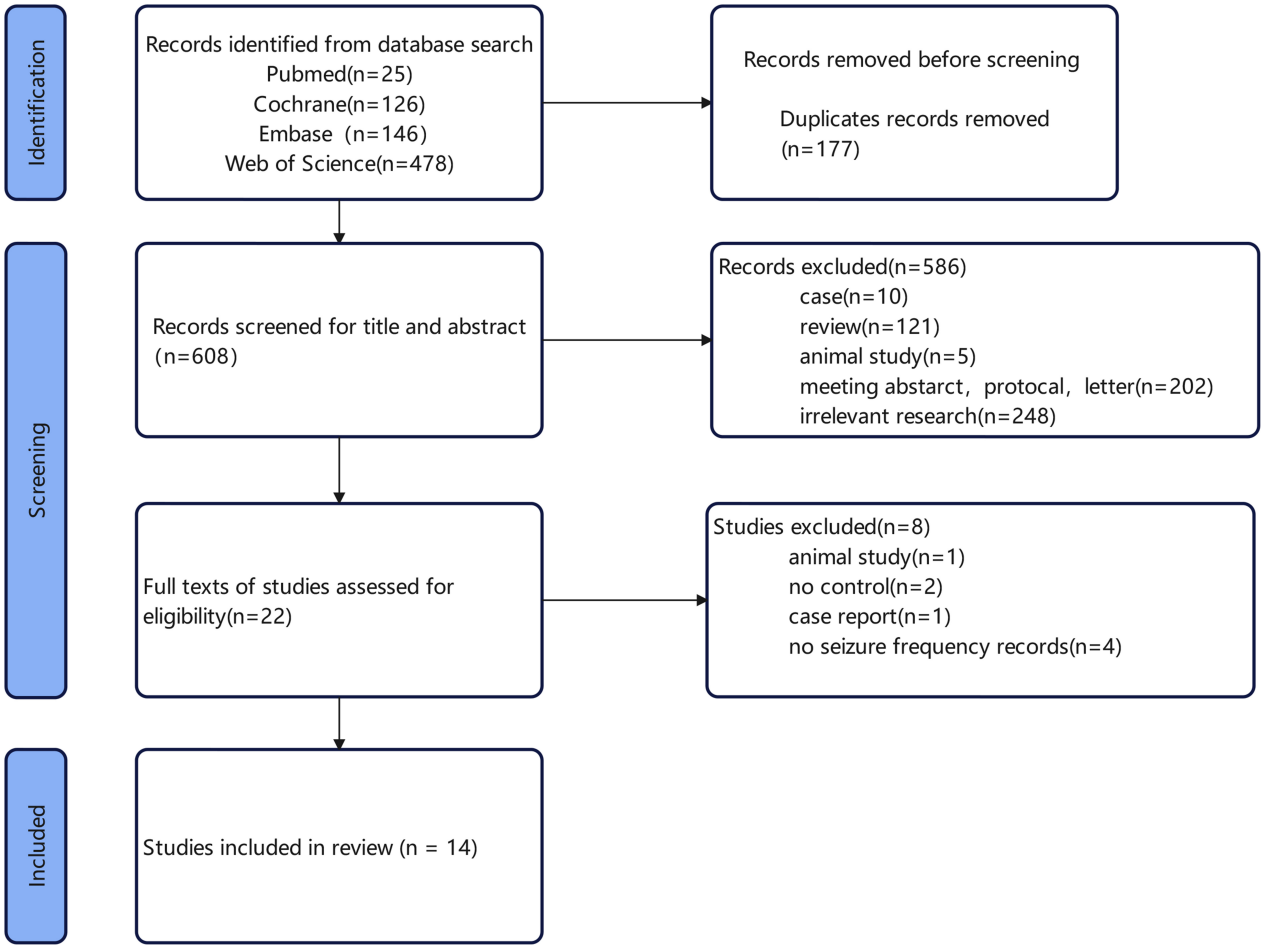
Flow chart of study selection process.

A total of 608 articles were retrieved. Specifically, 25 articles were identified through PubMed, 478 through Web of Science, 146 through Embase, and 126 through The Cochrane Library. After screening, 14 articles were included in the study. The flowchart of literature retrieval and results are shown in Figure 1. Among these 14 studies, 13 were randomized, double-blind trials, with four utilizing a crossover design. The remaining study was a randomized single-blind controlled trial. A total of 342 epilepsy patients were included in this meta-analysis, all of whom received catodal tDCS as the treatment modality. The basic characteristics of the included studies are summarized in Table 1. The follow-up time across the selected articles ranged from 1 week to 3 months (median, 1 month), with a notable frequency of 2-month follow-ups observed. We therefore selected 1 and 2 months for subgroup analysis. The treatment sessions were 1–14 (median, 5). Therefore, we used five sessions for subgroup analysis of cut-off value in the following analyses (median, five sessions).

**Table 1:** Basic characteristic and summary of included studies investigating efficacy in tDCS.

**References**	**Type and design**	**Number**	**Canode**	**Athode**	**Follow-up time**	**Stimulation sessions**	**Findings**	**Adverse effects**
Tecchio et al. (31)	Randomized double-blind crossover	6 TLE	The epileptic focus	The opposite homologous region	1 week	Two stimulation sessions	The one-session tDCS treatment did not reduce seizures, but it did affect brain connectivity.	
Assenza et al. (32)	Randomized double-blind crossover	10 TLE	The epileptic focus	The opposite homologous region	1 week	Two stimulation sessions	There were a significant reduction in seizure duration (71%) after tDCS treatment but not in IEDs	Skull itching is frequent in both tDCS (8/10) and sham group (7/10)
Zoghi et al. (69)	Randomized blind controlled trial	23 TLE	The affected temporal lobe	The contralateral supraorbital area	4 weeks	One session (9-20-9)	The use of tDCS reduced seizures by 42% in patients with refractory temporal lobe epilepsy and decreased cortical excitability as indicated by SICI measurements.	Itching sensations (2/10)
Fregni et al. (41)	Randomized double-blind controlled trial	19 MCD	The epileptic focus	A silent area free of ED	30 days	One session	There were significant reduction in EDs (64.3%), but not for clinical seizure frequency, also with a trend for long lasting effects of DC polarization on the cortical excitability.	Itching of the site of stimulation is few: 3/10 in tDCS group and 1/9 in sham group
Tekturk et al. (70)	Randomized double-blind cross-over study	12 mTLE	The affected HS side	The contralateral supraorbital region	2 months	3 sessions	The proportion of 50% responders was 83.33% and people achieving seizure freedom was 50% at the 2-month follow-up	Most patients report a tingling sensation
Luo et al. (37)	Randomized double-blind control trial	25 FE	The epileptic focus	A silent area free of ED	4 weeks	5 sessions	Cathodal tDCS didn't enhance cognitive performance and decrease seizure frequency. Cathodal tDCS can suppress EDs in 4 weeks after tDCS;the brain networks as assessed by small-worldness index(S) had a significant reduction after tDCS treatment.	One report sense of pricking; one report slight dizziness and one report seizure during treatment
Liu et al. (35)	Randomized double-blind controlled trial	33 TLE	The left DLPFC	The right supraorbital area	4 weeks	5 sessions	No significant improvements in seziure frequency, IEDs, working memory, verbal memory were noted after treatment. Mood assessed by NDDI-E and BDI improved after tDCS treatment while didn't persist to the 2- or 4-week follow-up.	Few side effects, including headache, pain, scalp burns, skin redness, sleepiness, concentration, acute mood change.
Mota et al. (36)	Randomized double-blind crossover trial	26 TLE	The right side of DLPFC	The left side of DLPFC	2 months	20 session	There was no statistically significant improvement in seizure frequency and mood performance following tDCS compared to control group.	
Rezakhani et al. (34)	Randomized double-blind controlled trial	20 FE	The epileptic focus		3 months	10 sessions	The treatment group showed a significant reduction in the frequency of seizure and IEDs at 2 weeks, 1 month, and 3 months after stimulation. Additionally, an increase in MoCA score was observed 2 weeks after the intervention.	7 of 11 in tDCS and 3 of 12 in sham group reported moderate or severe adverse effects including headache, itching sensation and burning sensation
Ashrafzadeh et al. (39)	Randomized double-blind controlled trial	18 FE	The most active interictal epileptiform discharge	Contralateral area	4 weeks	5 sessions	There was a significant reduction in seizure duration but not seizure frequency and IEDs after tDCS treatment	A mild itching sensation in 3/18 participants
San-Juan et al. (33)	Randomized double-blind three-arms trial	28 TLE	The most active epileptic focus	A silent supraorbital area	2 months	3 sessions or 5 sessions	There was a notable decrease in seizure frequency observed in the 3-session and 5-session active group at the 2-month (43.4 and 54.6%) of follow-up.	Mild itching (90%); Two report headache after treatment
Yang et al. (30)	Randomized double-blind three-arms trial	70 FE	Epileptogenic focus	A contralateral, silent, and relatively far area to the cathode	8 weeks	14 sessions	Patients with refractory focal epilepsy had significantly decreased SFs by 20-min and 2 × 20-min tDCS treatment for consecutive 14 days, with the latter benefitting the most.	Itching of the site of stimulation is frequent: 40/49 in tDCS group and 2/21 in sham group; five participants reported seizure during treatment; 3 in tDCS group and 2 in sham group
Auvichayapat et al. (42)	Randomized blind controlled trial	36 FE	Epileptogenic focus	Contralateral area	4 weeks	1 session	The active tDCS treatment led to immediate and not sustained reduction in IEDs, with a marginally but significant in seizure frequency observed 4 weeks later.	One developed a transient erythematous rash
Auvichayapat et al. (43)	Randomized, double-blind controlled trial	22 LGS	Left primary motor cortex	Contralateral shoulder	4 weeks	5 sessions	In the treatment group, both daily seizure frequency and IEDs significantly reduced at 4 and 3 weeks, respectively compared to the sham group.	One patient had three points of 1 mm superficial skin burn and completely resolved in 2 days

### 3.2 Quality assessment of included studies

**Figure 2:**
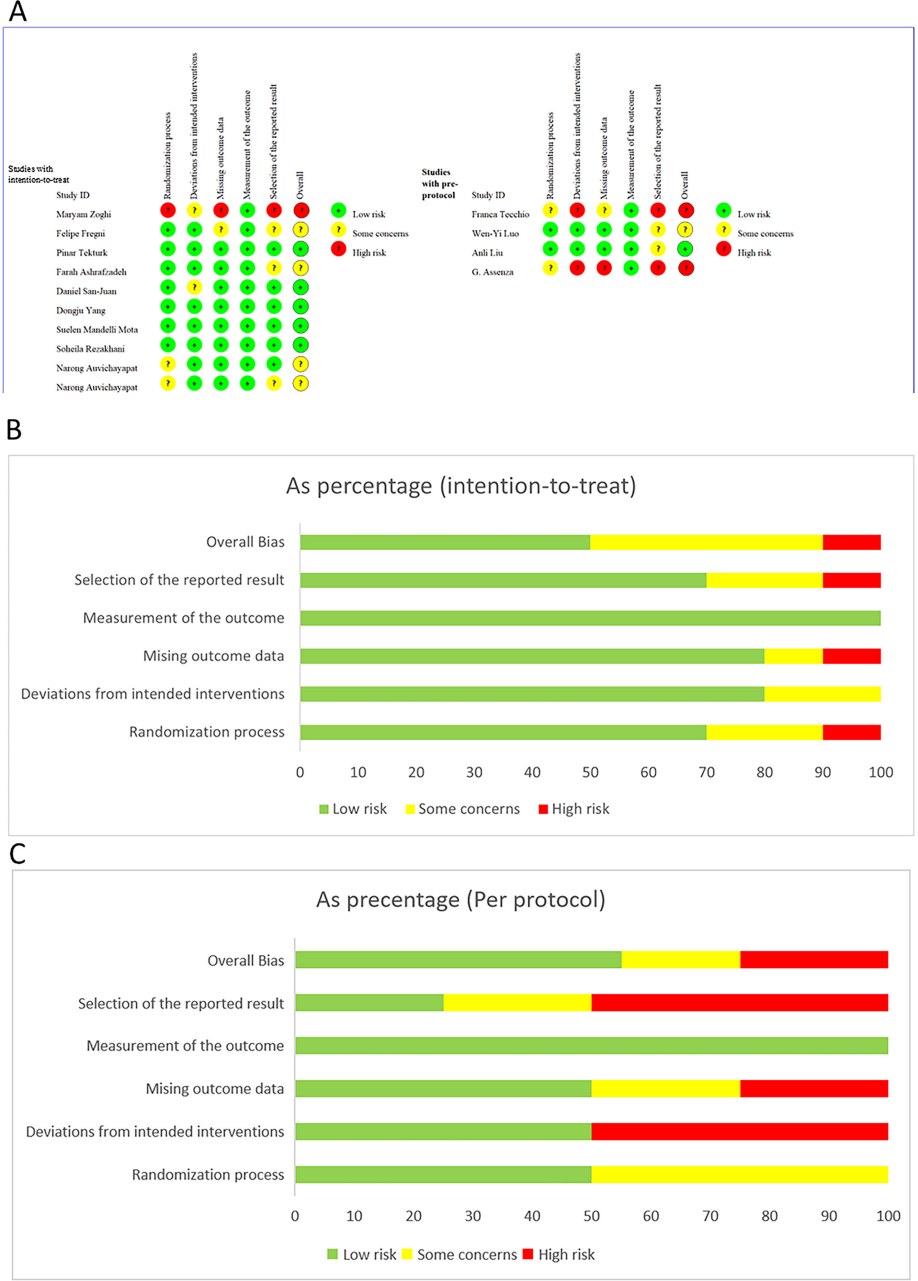
Risk of bias assessment according to the Cochrane collaboration's risk of bias assessment tool. **(A)** Assessment for 14 included studies, **(B)** assessment for studies with intention to treat design, **(C)** assessment for studies with per-protocal design.

Risk of bias assessment was conducted for the final 14 studies, and a methodological quality assessment summary figure (Figure 2) was generated using Excel software. According to ROB2.0 assessment, among the included studies, 50% exhibited low risk of bias in the intention-to-treat (ITT) analysis, while 10% showed high risk of bias in ITT analysis. For the per-protocol (PP) analysis, 55%, 20%, and 25% of studies displayed low risk, some concerns, and high risk of bias, respectively. Sources of high bias risk included baseline imbalance, improper handling of missing outcome data, and potential reporting biases. Overall, the included literature demonstrated relatively high quality. Detailed ROB2.0 results are presented in Figure 2.

### 3.3 Meta-analysis results

#### 3.3.1 Seizure frequency

**Figure 3:**
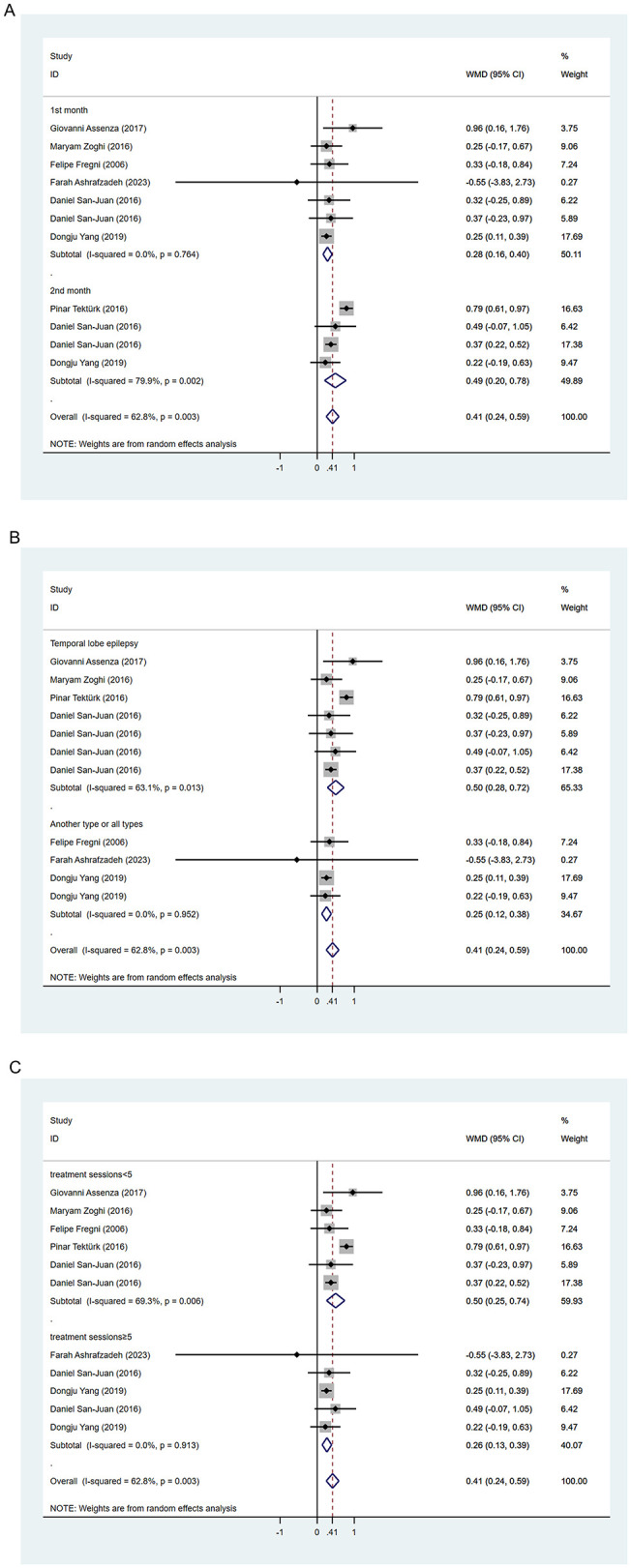
Forest plots of the effects of tDCS on the seizure reduction for **(A)** follow-up time, **(B)** seizure type, and **(C)** treatment session. WMD, Weighted Mean Difference.

**Figure 4:**
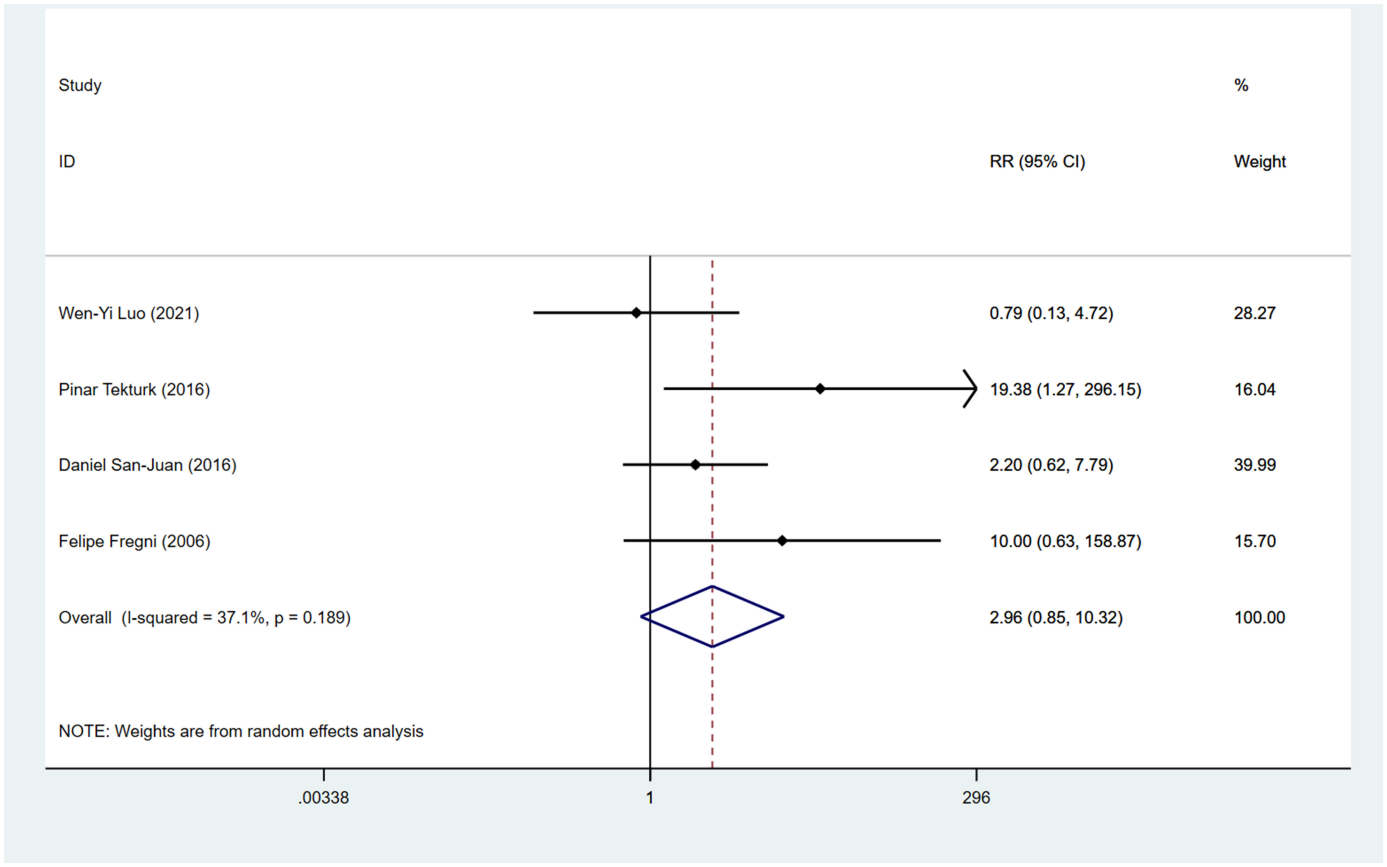
Forest plots of the effects of tDCS on the favorable response. RR, risk of ratio.

During the follow-up period, 64.3% (9/14) of the included studies reported statistically significant reduction in seizure frequency. Among these, Yang et al.'s study (30) was split into two articles due to different treatment durations, and Tecchio et al. (31) and Assenza et al. (32) were from the same study, with only the latter included in the meta-analysis covering all participants. We employed a random-effects model for data synthesis (*I*^2^ = 62.8%, *p* = 0.003). Overall results showed that tDCS treatment significantly reduced seizure frequency compared to sham stimulation after treatment (WMD 0.41, 95% CI 0.24, 0.59). Subgroup analyses were subsequently conducted to explore sources of heterogeneity. Two studies (30, 33) reported seizure frequency reduction at different time points after tDCS treatment. We found significant reduction in seizure frequency within 1 month post-tDCS treatment (WMD 0.28, 95% CI 0.16, 0.40) and sustained efficacy within 2 months' post-treatment (WMD 0.49, 95% CI 0.20, 0.78; Figure 3A). Treatment session duration (< 5 sessions vs. ≥5 sessions) and exclusively inclusion of temporal lobe epilepsy patients were not identified as sources of overall effect heterogeneity. However, shorter intervention duration (< 5 sessions; WMD 0.50, 95% CI 0.25, 0.74; Figure 3B) and temporal lobe epilepsy patients (WMD 0.50, 95% CI 0.28, 0.72; Figure 3C) showed greater reduction in seizure frequency. Four studies reported the proportion of participants achieving ≥50% reduction in seizures, indicating no significantly higher proportion among tDCS-treated patients compared to those receiving sham stimulation (RR 2.96, 95% CI 0.85, 10.32; Figure 4).

#### 3.3.2 Neuropsychological evaluation

Four studies utilized neuropsychological tests to assess changes in cognition and mood in epilepsy patients before and after tDCS treatment (34–37). Three studies employed the Montreal Cognitive Assessment (MoCA) or Mini-Mental State Examination (MMSE) to evaluate cognitive function comprehensively. Only Rezakhani observed an improvement in MoCA scores 2 weeks post-stimulation (34). However, this difference was not significant upon re-evaluation 1 month later, potentially due to a learning effect in MoCA testing. Across these studies, no statistically significant improvements or deteriorations in overall cognition were found with tDCS treatment. Liu et al. (35) further evaluated improvements in cognitive domains such as working memory and executive function post-treatment, with no significant improvements observed during the 1-month follow-up.

#### 3.3.3 fMRI and EEG results

Ten studies reported changes in electroencephalogram (EEG) before and after tDCS treatment. Four studies did not find significant changes in pre-ictal epileptic activity following tDCS treatment (35, 38–40). Six studies (33, 34, 37, 41–43) found a reduction in interictal epileptic-like activity post-treatment. Among these, Fregni observed patients with cortical dysplasia after treatment showed a trend toward reduced epileptic activity, albeit possibly only maintained for a few sessions (41). Rezakhani observed a reduction in interictal epileptic-like discharges 3 months post-treatment (36), while San-Juan's study showed a transient effect of reduced interictal epileptic-like discharges post-tDCS treatment (34). Luo et al. (37) used graph theory-based EEG analysis to study brain network characteristics in epilepsy, finding a decrease in small-worldness index post-tDCS treatment, suggesting refined information transfer efficiency, potentially inhibiting the propagation of epileptic-like discharges, although this study did not find a decrease in seizure frequency. Tecchio et al. (32) proposed functional magnetic resonance imaging (fMRI) findings in epilepsy patients post-tDCS treatment, indicating changes in functional connectivity in epileptic regions. Changes in functional connectivity values across all frequency bands were significantly correlated with seizure frequency, suggesting tDCS has the potential to disrupt pathological network activity in epilepsy patients.

#### 3.3.4 Adverse effects

Most adverse effects associated with tDCS are mild, with the most common being tingling sensations. Other frequently reported side effects include headache and burning sensations. These side effects are mostly self-limiting, transient, and tolerable. One participant withdrew from the study due to scalp burning sensation and pain. Two studies reported a total of five seizures (30, 37), which were brief focal seizures without additional intervention such as medication, and they were not directly attributable to stimulation, as these patients had a history of frequent seizures prior to treatment. Overall, tDCS appears to be safe for epilepsy treatment, though large-scale trials are needed for further validation.

### 3.4 Publication bias and sensitivity analysis

**Figure 5:**
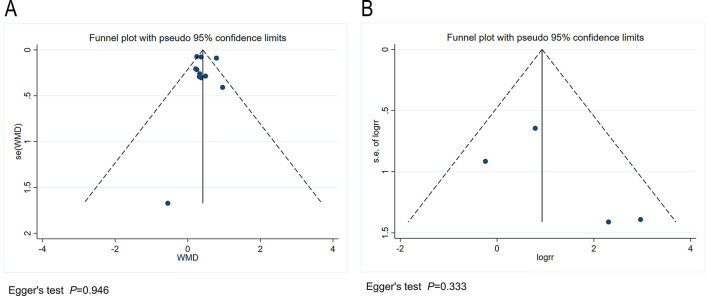
**(A)** Funnel plot for the publication bias regarding the seizure reduction compared with baseline. **(B)** Funnel plot for the publication bias regarding the responder rate.

**Figure 6:**
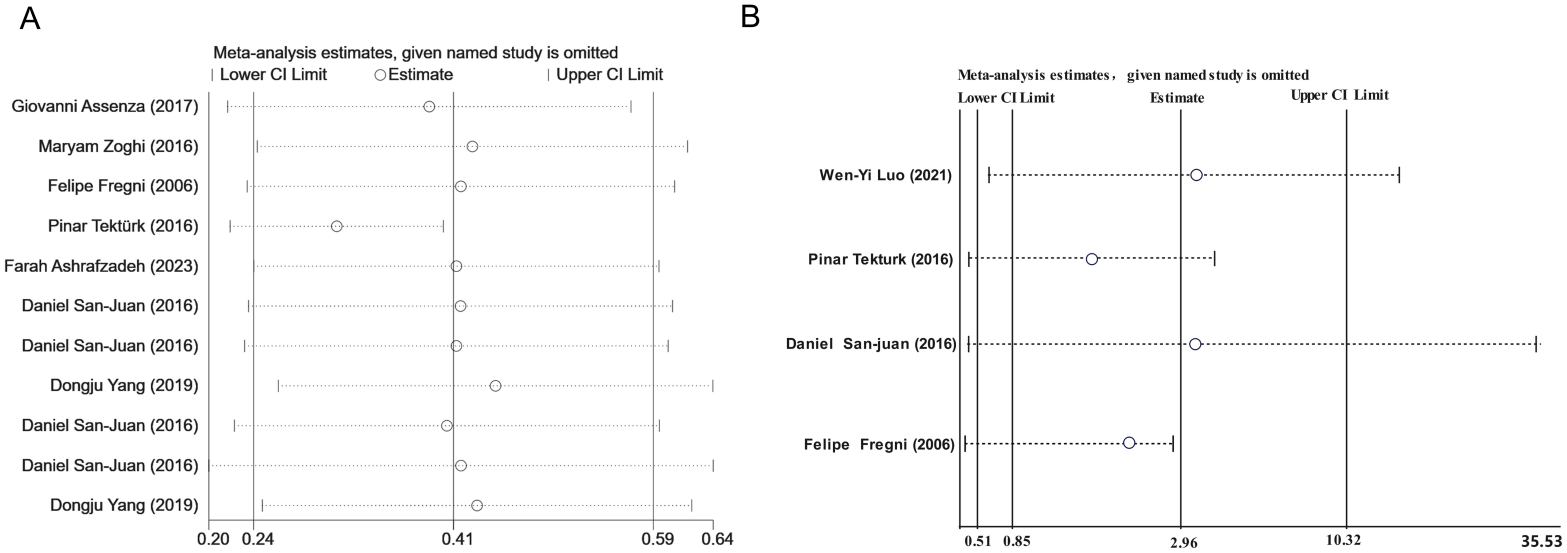
**(A)** Sensitivity analysis of seizure reduction compared with baseline. **(B)** Sensitivity analysis of responder rate.

We conducted a publication bias analysis for the included indicators. A funnel plot was used to visually present publication bias, and Egger's test was employed to analyze the funnel plot, with *p* > 0.05 indicating no publication bias (Figure 5). The results indicate no publication bias present across the included indicators. Sensitivity analysis was performed by individually excluding each study from the meta-analysis to rule out the excessive influence of any single study on the meta-analysis results. The sensitivity analysis results indicated that the meta-analysis results were stable and reliable (Figure 6).

## 4 Discussion

Despite several systematic reviews indicating (26, 44–46) that tDCS may reduce seizure frequency in the treatment of epilepsy, the findings regarding its effectiveness are inconclusive, and its efficacy has not been quantified. Here, we provided an updated meta-analysis of RCTs available to address this knowledge gap concerning the efficacy of tDCS in epilepsy treatment. We used seizure frequency reduction as the primary outcome to mitigate the influence of baseline differences on outcomes and employed WMD as the effect size measure for easier interpretation. Our results indicated that tDCS reduced seizure frequency by ~28 and 49% within 1- and 2-month follow-ups post-treatment, respectively. Further subgroup analysis found that it was more effective for participants with temporal lobe epilepsy. However, we did not find that it effectively increased the proportion of patients whose seizure frequency was reduced by 50% or more. On the other hand, consistent with systematic review findings, tDCS showed no clear improvement in cognitive and emotional functions.

To mitigate the heterogeneity caused by varying follow-up durations, this meta-analysis quantitatively analyzed changes in seizure frequency within 1 and 2 months intervals post-tDCS treatment. It observed a short-term reduction in seizure frequency following tDCS treatment, consistent with our clinical observations. Additionally, Kaufmann et al. prospectively included 15 patients with refractory focal epilepsy to explore the acute effects of tDCS, finding significant reduction in seizure frequency and epileptic-like discharges 48 h post-treatment, with the most pronounced effects observed 3–21 h post-stimulation (47). Initial placebo effects was large treated with brain stimulation (48, 49), potentially overestimating its immediate efficacy. In fact, traditional invasive neuromodulation techniques only achieve seizure frequency reduction of 14.7% (VNS), 32.1% (RNS), and 40.4% (DBS) within the first 3 months post-treatment (50). Nevertheless, leveraging placebo effects effectively could aid epilepsy patients and potentially synergize with other therapeutic mechanisms (49). The implementation of blinding closely correlates with placebo effects, with all included studies in our analysis employing blinding. However, few studies assessed the quality of blinding. Encouragingly, our results suggested that the efficacy within 2 months of treatment was more pronounced than within 1 month, contrasting with findings for TMS, which showed short-term effects in refractory epilepsy interventions but an increasing trend in seizure propensity over time with stimulation (51). Given the limited number of studies included for the 2-month follow-up, caution is warranted in interpreting the long-term effects of tDCS. Future studies should be designed to explore the time-course effects of tDCS.

Further subgroup analyses did not reveal any influence of treatment duration on efficacy. This may be due to several reasons: Firstly, the lack of dose-response relationship trials resulted in a less compressive categorization of treatment duration. Secondly, this observation might suggest that tDCS was effective in specific populations, such as mesial temporal lobe epilepsy and Lennox-Gastaut syndrome (52). For instance, both shorter (< 5 sessions) and longer (>5 sessions) treatments have been found effective for medial temporal lobe epilepsy, consistent with quantitative analysis results targeting temporal lobe epilepsy, possibly due to the specific treatment targets of this epilepsy syndrome. Abuhaib et al. stimulated the FT7 area in patients with temporal lobe epilepsy and used magnetic resonance spectroscopy to find a decrease in γ-aminobutyric acid (GABA) in the temporal region, which was related to interictal epileptic discharges (53). Compared to repetitive transcranial magnetic stimulation (rTMS), tDCS may have better efficacy for temporal lobe epilepsy, while the latter may be more effective for neocortical epilepsy (54). Although the comparison of these two was not the focus of this study, it suggested the need for future studies to include a more diverse range of epilepsy syndromes and explore other relevant biomarkers besides electroencephalography.

Through systematic review, we have preliminarily explored the mechanisms of tDCS, which may act by inhibiting cortical excitability, modulating cortical excitation/inhibition balance, and reshaping epilepsy networks. Four studies have reported the frequency of IED showed no significant change following tDCS stimulation when compared to sham groups. Among these, three studies reported no reduction in seizure frequency as well. Equally, six studies reported IED frequency decrease after treatment, with four of them reported seizure reduction. The observed decrease in IEDs post-treatment can possibly be a biomarker linked to favorable treatment response. In addition, the results in IED change after tDCS stimulation differed both in shorter and longer treatment. Recent study explored the immediate effects of tDCS, using stereo-electroencephalography (SEEG) recordings to accurately localize epileptogenic zone (55). One finding is that IED changes were also inconsistent after shorter treatment (one session, 20 min). Importantly, one patient after sham stimulation reported significant IED decrease and they pointed out most patients had no insufficient IED for statistical analysis as well as inter-subject variability in baseline IEDs, and Fregni reported the number of IEDs were largely variable among patients accordingly. More uniform and less variable markers linked to brain response are required in the future.

Despite previous studies reporting that slow oscillatory transcranial direct current stimulation (so-tDCS) improves memory consolidation during naps in temporal lobe epilepsy patients (56), and high-definition transcranial direct current stimulation (HD-tDCS) enhances attention and working memory in frontal lobe epilepsy patients (57, 58). Further systematic review results suggest no significant improvement in cognition with tDCS. This may be attributed to several reasons: Firstly, in terms of treatment sites, the dorsolateral prefrontal cortex is a common target for treating cognitive impairments (59–62). In three studies assessing cognition as an outcome, two used cathodal electrode stimulation at epileptic foci, and the studies utilized modified versions of tDCS, such as so-tDCS aiming to simulate slow waves during sleep, enhancing endogenous slow oscillatory activity (63), while HD-tDCS offers advantages in terms of current concentration (64). Secondly, there is a lack of analysis on cognition domains with included studies using MOCA and MMSE, which may have lower sensitivity to evaluate tDCS treatment effects. Thirdly, small samples in these three articles may result in insufficient statistical power, leading to type II statistical errors and false-negative results.

Limitations of this work include the recent emergence of tDCS as a treatment modality for epilepsy, which has not yet advanced to the stage of large-scale randomized controlled trials, resulting in small sample sizes. Additionally, inavailability to clinical data related to seizure frequency in some studies has limited the reliability of our quantitative analysis conclusions. In terms of heterogeneity, existing studies have employed various stimulation parameters (e.g., stimulation duration, current intensity, stimulation intervals, and electrode size), all of which may influence the post-treatment effects of tDCS, potentially even reversing its excitatory and inhibitory effects (65, 66). Moreover, factors such as participants' medication use, seizure frequency, and different etiologies of epilepsy could be underlying sources of heterogeneity in the studies. For instance, cathodal tDCS enhances GABA-mediated intracortical inhibition (67), while NMDA receptor antagonists may inhibit the effects of cathodal tDCS post-treatment (68).

Future research should focus on several key areas: Firstly, conducting larger-scale multicenter randomized controlled trials to enhance the reliability and generalizability of results. Secondly, systematically studying the impact of different stimulation parameters on efficacy to identify optimal stimulation protocols. Additionally, long-term follow-up studies are needed to understand the sustained effectiveness and potential side effects of tDCS. Lastly, as neuromodulation becomes increasingly important in the coming years, research should particularly focus on identifying specific populations that can benefit from various neuromodulation techniques beyond tDCS, and there is a need for more cost-effectiveness studies to guide treatment decisions and resource allocation. These studies will contribute to optimizing the application strategies of neuromodulation and improving its practical effectiveness in epilepsy treatment.

## 5 Conclusion

This study demonstrates that tDCS significantly reduces seizure frequency post-treatment. Future research should include longer-term and larger RCTs, as well as other cohort studies, to validate the findings of this study.

## Data Availability

The original contributions presented in the study are included in the article/Supplementary material, further inquiries can be directed to the corresponding authors.
