# Supplementary material for: Transcranial direct current stimulation in the management of epilepsy: a meta-analysis and systematic review
Source: Front Neurol. 2024 Nov 11;15:1462364. doi: 10.3389/fneur.2024.1462364 (PMC11586187; doi:10.3389/fneur.2024.1462364)
Supplement: Supplementary file 1 [file Data_Sheet_1.docx]

Search Strategy

PubMed

| No. | Query | Result |
| --- | --- | --- |
| #1 | ((((Transcranial Direct Current Stimulation[Title/Abstract]) OR (tDCS[Title/Abstract])) OR (t-DCS[Title/Abstract])) OR (brain polarization[Title/Abstract])) OR (galvanic stimulation[Title/Abstract]) | 8,492 |
| #2 | Transcranial Direct Current Stimulation [MeSH Terms] | 5,407 |
| #3 | (((((Transcranial Direct Current Stimulation[Title/Abstract]) OR (tDCS[Title/Abstract])) OR (t-DCS[Title/Abstract])) OR (brain polarization[Title/Abstract])) OR (galvanic stimulation[Title/Abstract])) OR (Transcranial Direct Current Stimulation[MeSH Terms]) | 9,584 |
| #4 | (((epilepsy[MeSH Terms]) OR (epilepsy[Title/Abstract])) OR (Epilep*[Title/Abstract])) OR (seizur*[Title/Abstract]) | 258,320 |
| #5 | Randomized controlled trial[Publication Type] OR controlled clinical trial [Publication Type] random*[Title/Abstract] OR placebo[Title/Abstract] | 632,192 |
| #6 | (((((((Transcranial Direct Current Stimulation[Title/Abstract]) OR (tDCS[Title/Abstract])) OR (t-DCS[Title/Abstract])) OR (brain polarization[Title/Abstract])) OR (galvanic stimulation[Title/Abstract])) OR (Transcranial Direct Current Stimulation[MeSH Terms])) AND ((((epilepsy[MeSH Terms]) OR (epilepsy[Title/Abstract])) OR (Epilep*[Title/Abstract])) OR (seizur*[Title/Abstract]))) AND (randomized controlled trial[Publication Type] OR controlled clinical trial [Publication Type] random*[Title/Abstract] OR placebo[Title/Abstract]) | 25 |

Embase

| No. | Query | Result |
| --- | --- | --- |
| #1 | transcranial AND direct AND current AND ('stimulation'/exp OR stimulation) | 14,141 |
| #2 | 'transcranial direct current stimulation':ab,ti OR tdcs:ab,ti OR 't dcs':ti,ab,kw OR 'brain polarization':ab,ti OR 'galvanic stimulation':ab,ti | 11,698 |
| #3 | #1 OR #2 | 15,410 |
| #4 | epilepsy:ab,ti OR epilep*:ab,ti OR | 351,553 |
| #5 | 'epilepsy'/exp OR epilepsy | 343,623 |
| #6 | #4 OR #5 | 433,445 |
| #7 | 'randomized controlled trial':it OR 'controlled clinical trial':it OR random*:ab,ti OR placebo:ab,ti | 2,182,176 |
| #8 | #3 AND #6 AND #7 | 146 |

Web of Science

| No. | Query | Result |
| --- | --- | --- |
| #1 | Transcranial Direct Current Stimulation (Topic) OR tDCS (Topic) OR t-DCS (Topic) OR brain polarization (Topic) OR galvanic stimulation (Topic) and Preprint Citation Index (Exclude – Database) | 26,875 |
| #2 | epilepsy (Topic) OR Epilep* (Topic) OR seizur* (Topic) and Preprint Citation Index (Exclude – Database) | 486,538 |
| #3 | random* (Abstract) OR Epilep* (Abstract) OR randomized controlled trial (Abstract) OR placebo (Abstract) and Preprint Citation Index (Exclude – Database) | 15,410 |
| #4 | #1 AND #2 AND #3 and Preprint Citation Index (Exclude – Database) | 478 |

Cochrane Date

| No. | Query | Result |
| --- | --- | --- |
| #1 | epilepsy | 26,875 |
| #2 | (epilepsy):ab，ti，kw or (Epilep*):ab，ti，kw or (seizur*):ab，ti，kw | 12825 |
| #3 | Transcranial Direct Current Stimulation | 6608 |
| #4 | (Transcranial Direct Current Stimulation):ab，ti，kw or (tDCS):ab，ti，kw or (t-DCS):ab，ti，kw or (brain polarization):ab，ti，kw or (galvanic stimulation):ab，ti，kw | 6503 |
| #5 | #1 or #2 | 14613 |
| #6 | #3 or #4 | 7315 |
| #7 | (randomized controlled trial):pt OR (controlled clinical trial):pt OR (random*):ti,ab,kw OR (placebo):ti,ab,kw (Word variations have been searched) | 1442876 |
| #8 | #5 and #6 and #7 | 265 |
